# WormGender – Open-Source Software for Automatic *Caenorhabditis elegans *Sex Ratio Measurement

**DOI:** 10.1371/journal.pone.0139724

**Published:** 2015-09-30

**Authors:** Marta K. Labocha, Sang-Kyu Jung, Boanerges Aleman-Meza, Zheng Liu, Weiwei Zhong

**Affiliations:** Department of BioSciences, Rice University, Houston, Texas, United States of America; East Carolina University, UNITED STATES

## Abstract

Fast and quantitative analysis of animal phenotypes is one of the major challenges of current biology. Here we report the WormGender open-source software, which is designed for accurate quantification of sex ratio in *Caenorhabditis elegans*. The software functions include, i) automatic recognition and counting of adult hermaphrodites and males, ii) a manual inspection feature that enables manual correction of errors, and iii) flexibility to use new training images to optimize the software for different imaging conditions. We evaluated the performance of our software by comparing manual and automated assessment of sex ratio. Our data showed that the WormGender software provided overall accurate sex ratio measurements. We further demonstrated the usage of WormGender by quantifying the high incidence of male (*him*) phenotype in 27 mutant strains. Mutants of nine genes (*brc-1*, *C30G12*.*6*, *cep-1*, *coh-3*, *him-3*, *him-5*, *him-8*, *skr-1*, *unc-86*) showed significant *him* phenotype. The WormGender is written in Java and can be installed and run on both Windows and Mac platforms. The source code is freely available together with a user manual and sample data at http://www.QuantWorm.org/. The source code and sample data are also available at http://dx.doi.org/10.6084/m9.figshare.1541248.

## Introduction

One major challenge in modern biology is automation for high-throughput data acquisition and analysis. *Caenorhabditis elegans* is one of the model organisms widely used in genetic, developmental and neurobiological studies [1]. Application of RNA interference (RNAi) by easy protocols such as feeding [2, 3] enabled large-scale inactivation of genes in *C*. *elegans*, and fundamentally reversed the bottleneck of genetics from gene inactivation to phenotyping. Most early studies relied on manual observation for phenotyping, thus limiting their scopes to qualitative and simple phenotypes [4, 5, 6, 7, 8].

Various imagining systems have been developed to enable fast acquisition and quantitative analysis of multiple *C*. *elegans* phenotypes, such as locomotion [9, 10, 11, 12, 13, 14, 15], lifespan [16, 17], and embryonic count [18]. One of the most comprehensive *C*. *elegans* phenotyping systems was recently developed in our laboratory, enabling high-throughput analysis of multiple phenotypes, such as locomotion, lifespan, body size and egg-laying patterns [19].

One of the *C*. *elegans* phenotypes that have been widely studied is abnormal sex ratio. *C*. *elegans* have two sexes, hermaphrodites and males. Males are generally very rare in wild-type *C*. *elegans* [20]. They naturally arise as a result of sporadic chromosome-nondisjunction events during meiosis, and occur at a frequency of less than one male per 500 hermaphrodites under standard laboratory conditions [21]. There are many known mutations causing the phenotype of high incidence of males (*him*). They usually arise due to faulty chromosome segregation, sex determination, or dosage compensation [21, 22]. Mutations leading to the *him* phenotype are frequently associated with genes whose human orthologs play a role in important processes such as cancer development [23]. Therefore, it is crucial to study sex ratio in *C*. *elegans*. However, there is no automatic phenotyping system for such an important phenotype.

Here we present WormGender, an open-source software system designed for accurate and fast quantification of the *him* phenotype in *C*. *elegans*. We verified the performance of this software by comparing manual and automated counts of males and hermaphrodites, demonstrating that WormGender can provide accurate estimates of male percentage in a population. WormGender can be downloaded from http://www.quantworm.org/ or http://dx.doi.org/10.6084/m9.figshare.1541248.

## Methods

### Animals

The following strains were used: N2 (wild-type), CB1256 [*him-3(e1256)*], CB1416 [*unc-86(e1416)*], CB1489 [*him-8(e1489)*], CB4088 [*him-5(e1490)*], CB5380 [*fox-1(e2643)*], CV138 [*sgo-1(tm2443)*], DW102 [*brc-1(tm1145)*], JK3101 [*fbf-2(q738)*], MH801 [*sur-7(ku119)*], MT1080 [*sdc-1(n485)*], MT1446 [*her-1(n695)*], MT14851 [*set-2(n4589)*], MT2244 [*sel-10(n1077)*], SP488 [*smk-1(mn156)*], WWZ239 [*gpr-2(ok1179)*], WWZ241 [*klp-10(ok704)*], WWZ242 [*cki-2(ok2105)*], WWZ243 [*skr-1(ok1696)*], WWZ246 [*C30G12*.*6(ok2389)*], WWZ248 [srgp-1(ok300)], WWZ250 [*F52H3*.*4(ok2692)*], WWZ251 [*T08D2*.*7(ok431)*], WWZ252 [*zhp-3(ok1993)*], WWZ255 [*hcp-2(ok1757)*], WWZ256 [*coh-3(gk112)*], WWZ258 [*W02D9*.*3(ok2857)*], XY1054 [*cep-1(lg12501)*]. Strains were obtained from the Caenorhabditis Genetics Center (CGC) except for WWZ239, WWZ241, WWZ242, WWZ243, WWZ246, WWZ248, WWZ250, WWZ251, WWZ252, WWZ255, WWZ256, WWZ258, which were created by outcrossing six times the CGC strains RB1150, RB866, RB1692, VC1241, RB1846, RB570, RB2034, RB655, RB1620, RB1492, VC131, RB2143, respectively. Animals were grown at 20°C on standard nematode growth media (NGM) seeded with the OP50 strain of *Escherichia coli* as described [24].

### Imaging hardware

The imaging hardware included a dissecting microscope (SZ61, Olympus America, Center Valley, PA), motorized XY stage (H105 ProScan, PRIOR Scientific, Rockland, MA), stage control module (ProScan II Controller, PRIOR Scientific, Rockland, MA), and digital camera (Fire-i 501b, Unibrain, San Ramon, CA).

### Image acquisition

For image acquisition, synchronized adult animals were transferred onto 6-well scanning plates (modified NGM plates that do not contain peptone or cholesterol) using the following method. 1 ml S Basal solution (0.1M NaCl, 0.05M KH_2_PO_4_, 0.05M K_2_HPO_4_) was added to each well of the 6-well plates where animals were cultured. The liquid with animals was transferred to glass test tubes using Pasteur pipettes. The glass test tubes were placed on ice to let the animals settle at the bottom. Most of the liquid was then aspirated and the remaining liquid with worms was dropped with Pasteur pipettes onto scanning plates. The scanning plates were left air dry without lids for about 30 minutes. Animals were then killed by adding 10μl of 1M sodium azide to each well. The plates were then scanned using the QuantWorm imaging system [19] to obtain tiled images of worm plates with magnification set to reach a resolution of 6 to 7 μm/pixel.

### Image processing and analysis

**Fig 1:**
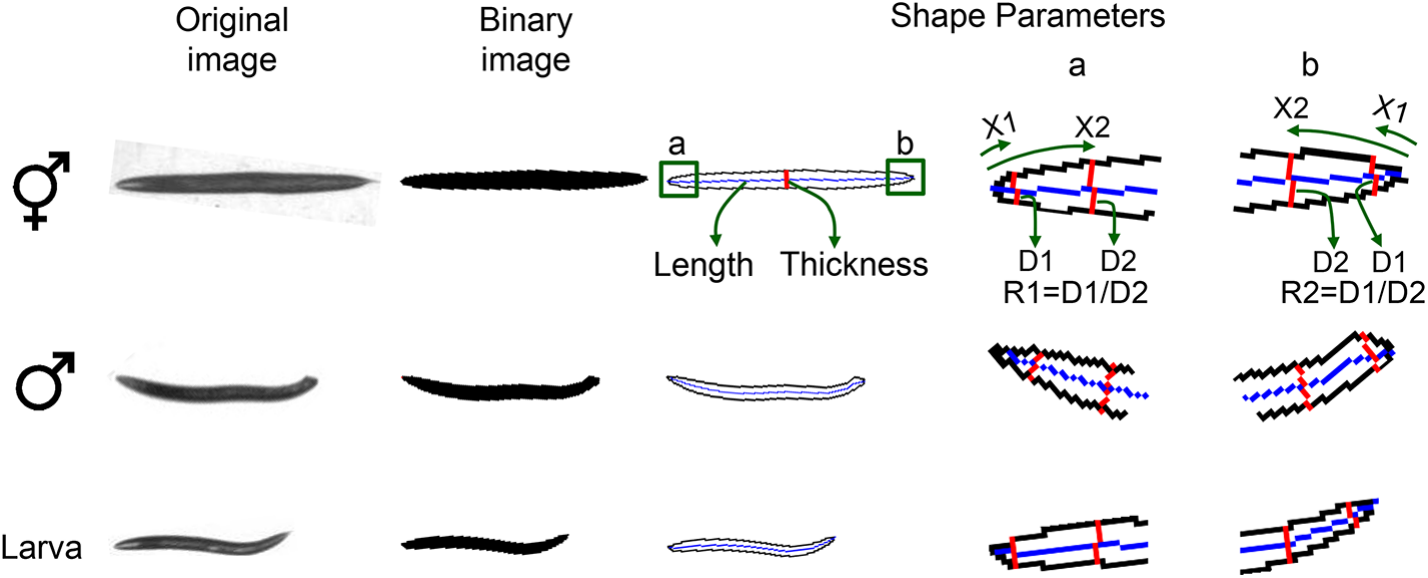
Shape parameters. Original image is binarized and skeletonized. Four shape parameters are calculated. Worm length is the length of the midline; worm thickness is defined as worm area divided by worm length; R1 and R2 are the ratios of worm diameters at two specific locations (X1 and X2) near the ends of the worm. Images labelled a and b are zoomed-in views of worm head and tail, respectively.

A large assembled image is created for each worm plate by stitching multiple tiled photos. The assembled image is binarized by adaptive local thresholding [25]. Region extraction is then applied to detect worm objects (Fig 1). Any invalid objects that are not worms are excluded from further image analysis. For example, tiny objects that are smaller than typical worms are screened out based on the bounding box size and area. To measure worm length, a skeleton curve is created from the binary worm image. Worm thickness is obtained by dividing the worm area (in pixel counts) by the length of the skeleton curve (in pixel counts). Using a micrometer per pixel ratio, actual length (μm) and worm thickness (μm) are computed. In addition to length and thickness, WormGender analyzes another two shape parameters, R1 and R2 (Fig 1), which are defined as the ratio of worm diameters (D1 and D2) at two specific locations (X1 and X2) on the skeleton curve at each end of the worm. X1 and X2 are empirically determined. After testing our images, we found that the optimal positions to measure diameter ratio is at 20 μm and 120 μm from the end (i.e., X1 = 20 μm and X2 = 120 μm in Fig 1). R2 is designated to be the larger value. R1 and R2 are used to detect the differences in tail shape between genders.

### Training set

WormGender requires a training set of images with known classifications of hermaphrodites, males, and larvae. The training set we used was supplied in the package as a default training set. However, users can replace that with a new one. Our training data set consists of 131 individual pictures of worms: 46 hermaphrodites, 46 males, and 39 larvae.

### Gender determination

WormGender first determines the shape parameter values from the training set. It then analyzes the parameter values from a new image, computes the Mahalanobis distance [26] between the parameter values of the unknown worm and each of the known classes (males, hermaphrodites, larvae) in the training set, and assigns the unknown worm to the class with the shortest Mahalanobis distance.

### User interface

**Fig 2:**
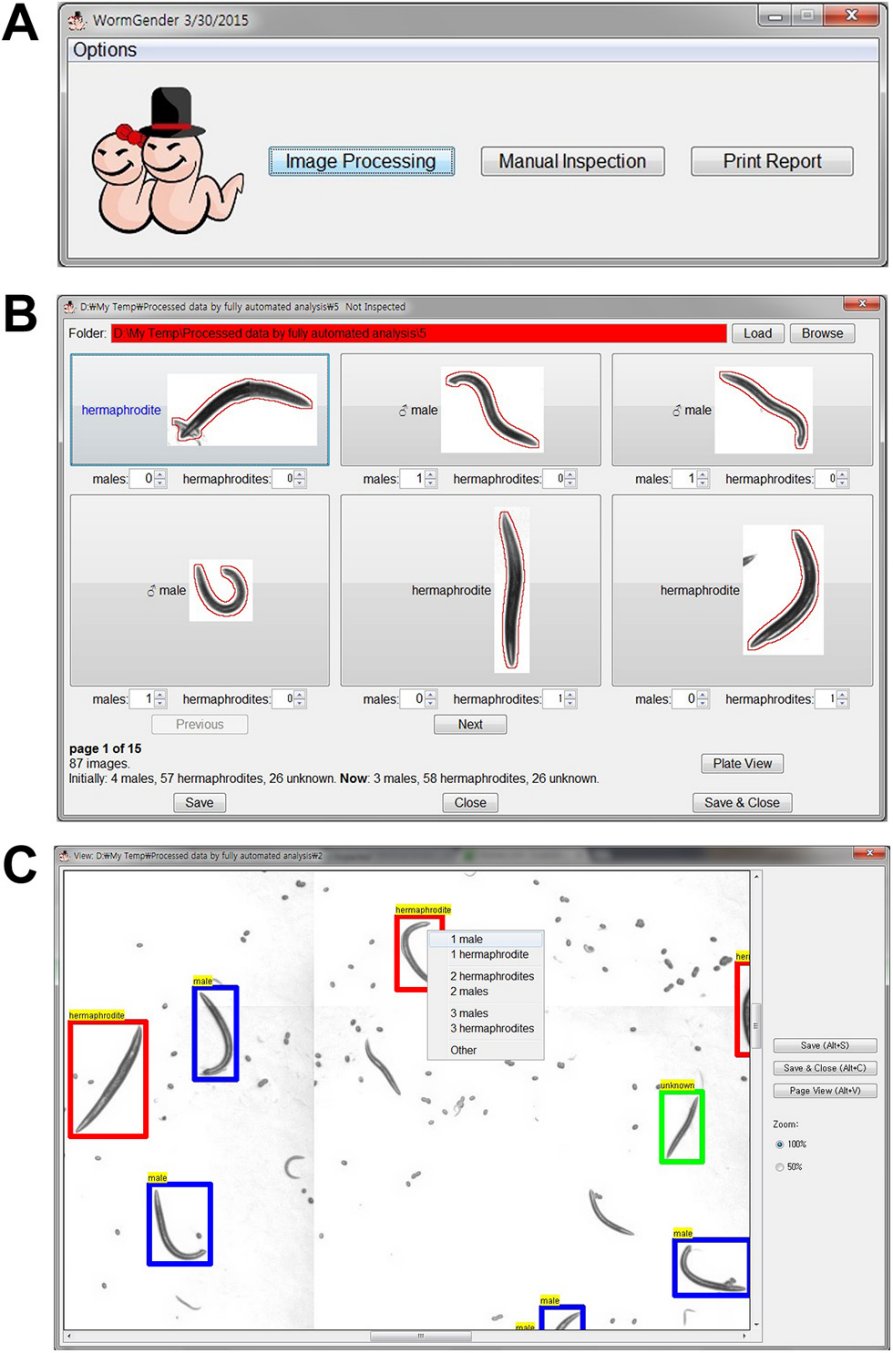
WormGender user interface. (A) WormGender has three command buttons: ‘Image Processing’ to conduct batch image processing, ‘Manual Inspection’ to review and correct computer analysis, and ‘Print Report’ to create a summary report file. (B) Clicking the ‘Manual Inspection’ button opens the manual inspection window, where a user can modify the classification of each worm when needed. (C) A user can also conduct manual inspection using the plate view.

A graphic user interface is designed for WormGender (Fig 2). A detailed user manual is available at http://www.quantworm.org/.

### Comparing manual and automatic counts

For data comparing manual and automatic counts, worm populations with different number of animals and various sex ratios were prepared using either of the following methods. The first method followed standard culture conditions. CB1256 [*him-3(e1256)IV*] worms were synchronized by bleaching gravid adults as described [24]. Eggs were cultured in M9 buffer with 5 μg/ml cholesterol overnight to obtain synchronized L1 larvae. L1s were dropped at the amount of 100, 150, 200, 250, 300, and 350 animals per well onto 6-well NGM plates seeded with OP50. Animals were grown for 3 days at 20°C to reach adulthood.

The second method followed RNAi conditions [27]. RNAi plates (NGM plates with 50μg/ml Carbenicillin and 1mM IPTG) seeded with the *E*. *coli* HT115 were used. Five L4 larvae of CB1489 [*him-8(e1489)IV*], or XY1054 [*cep-1(lg12501) I*] worms were picked onto each well of 6-well plates, cultured at 25°C for 24 hours, and removed. The remaining eggs on the plates were cultured for two days at 25°C to reach adulthood.

Animals were transferred to scanning plates and scanned. The images were analyzed using WormGender to obtain automatic counts. The plates were then examined manually under a dissecting microscope. Manual counts were obtained by aspirating individual worms.

For data on sex-ratio of different worm strains, the RNAi culture condition was used, and manual counts were conducted on experimental trials independent of automatic counts.

## Results and Discussion

### Classifying worms based on four shape parameters

Male worms are smaller with blunt tail ends. To capture the morphological differences between males and hermaphrodites, we used length and thickness to measure animal size, and diameter ratios at the ends (R1, R2) to measure tail shape (Fig 1).

**Fig 3:**
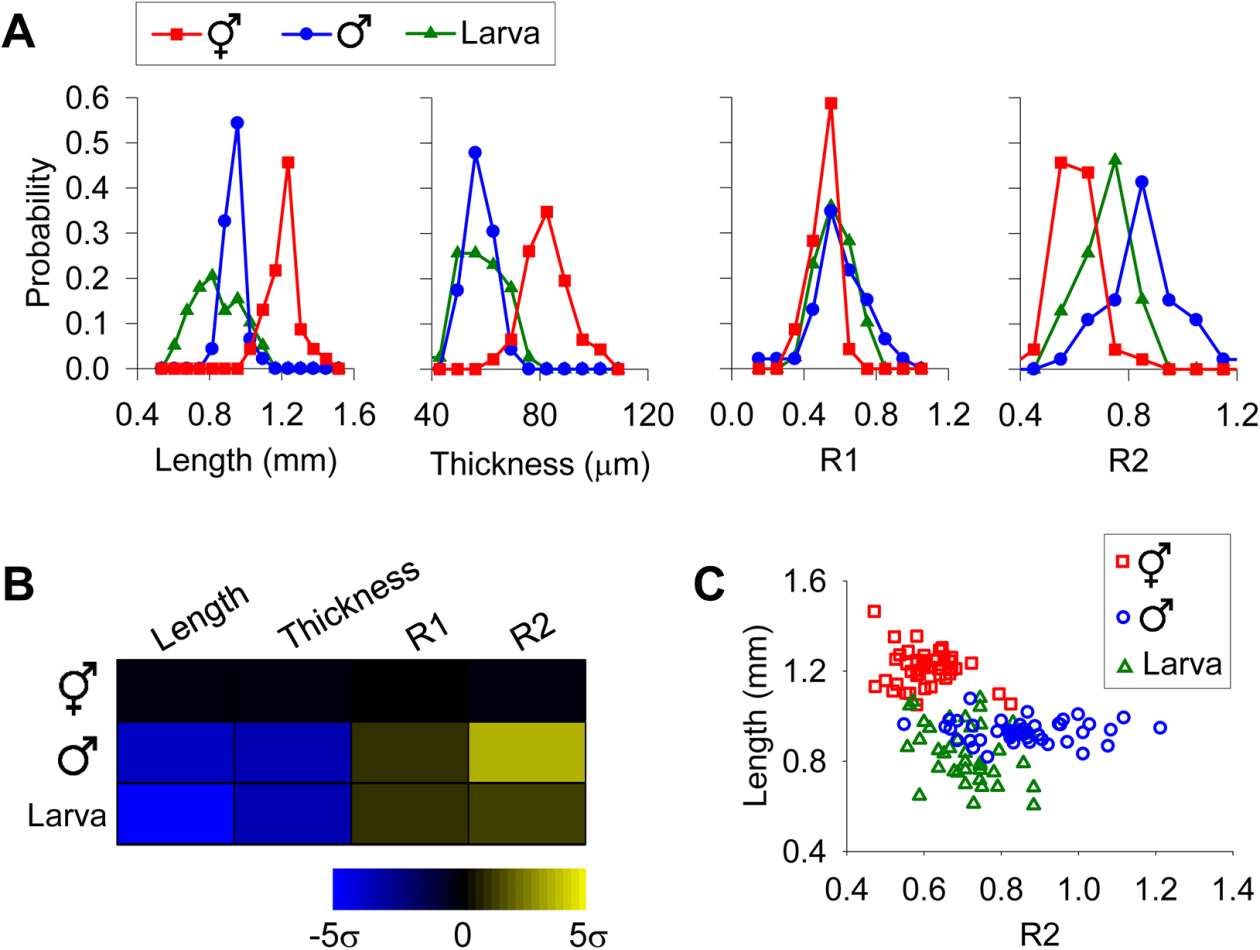
Using shape parameters to distinguish hermaphrodites, males and larvae. Images of 131 worms in three classes (46 hermaphrodites, 46 males, 39 larvae) were analyzed for the shape parameters. Raw data are listed in S1 Table. (A) Distribution of shape parameters in each worm class. (B) Phenotypic profiles using mean values of shape parameters. Z-scores were used to construct the heat map, displaying differences between mean values from a given class and hermaphrodites in multiple standard deviations. σ, standard deviation. (C) Using length and R2 to distinguish three different worm classes.

These four shape parameters, length, thickness, R1 and R2, are sufficient to distinguish males, hermaphrodites and young larvae. A significant difference was found between every pairs of worm classes in length and R2 (*p* < 0.01, one-way ANOVA and Scheffé *post hoc* analysis; Fig 3A). Hermaphrodites also showed significant difference from the other two worm classes in thickness and R1 (*p* < 0.05, one-way ANOVA and Scheffé *post hoc* analysis; Fig 3A). A phenotypic profile using the mean values of these four parameters showed distinctions among different populations (Fig 3B). While R2 was the most powerful parameter for gender determination (Fig 3A and 3B), including other parameters (e.g., length) improved the accuracy of classification (Fig 3C).

Because worm length and thickness are used as parameters, mutants with body size defects such as dumpy worms may not be correctly recognized if the training set is derived from wild-type worms. Similarly, because WormGender relies on shape measurements, animals with cuticle blisters will not be correctly recognized by the software. In addition, shape parameters cannot be extracted from clumped animals. Therefore, animals with aggregating behaviours must be immobilized on the scanning plates before they cluster.

Precise measurements of shape parameters require good image quality. Thick bacterial lawn with tracks can interfere with the image quality. Transferring worms to scanning plates can solve this issue. It is also important to use synchronized populations for WormGender. Ideally the worms should be young adults before they start to lay many eggs. Too many eggs on the image can interfere with the shape measurement. For example, if eggs are touching a hermaphrodite at the end of the body, the program may erroneously recognize the animal as a male.

### WormGender performance

To evaluate the performance of WormGender, we examined its speed and its accuracy. WormGender showed a good image processing speed: It took on average 30 seconds for WormGender to process the image of one well in a 6-well plate.

To evaluate the accuracy of WormGender, we first tested it in a controlled simulation. We split our training set of 46 hermaphrodites, 46 males and 39 larvae into two groups. We used one group as training set and the other as testing set (Experiment 1 in Table 1), and then switched the roles of the two groups and tested again (Experiment 2 in Table 1). In both simulated experiments, WormGender performed better in hermaphrodite and male recognition than larva recognition (Table 1). The most common WormGender classification errors included hermaphrodites misclassified as males, males misclassified as larvae, and larvae misclassified as either males or hermaphrodites (Table 1).

**Table 1:** WormGender accuracy in simulated experiments.

	Experiment 1			Experiment 2		
	Actual			Actual		
Classified as	Hermaphrodite	Male	Larva	Hermaphrodite	Male	Larva
Hermaphrodite	22 (96%)	0	3 (16%)	21 (91%)	0	0
Male	1 (4%)	21 (91%)	2 (10%)	2 (9%)	20 (87%)	4 (21%)
Larva	0	2 (9%)	14 (74%)	0	3 (13%)	15 (79%)

As most errors involve larvae, it is thus important to use synchronized adult population to minimize errors. Only L4 larvae were difficult for WormGender to recognize. Younger larvae did not affect WormGender performance because of drastic length differences between these small larvae and males and hermaphrodites. While using aged adult populations can further minimize the presence of L4 larvae, the presence of too many eggs produced by the adults can interfere with image quality. Empirically we found that using day-one adults seemed to reach a good balance between the two factors for most strains we tested.

**Fig 4:**
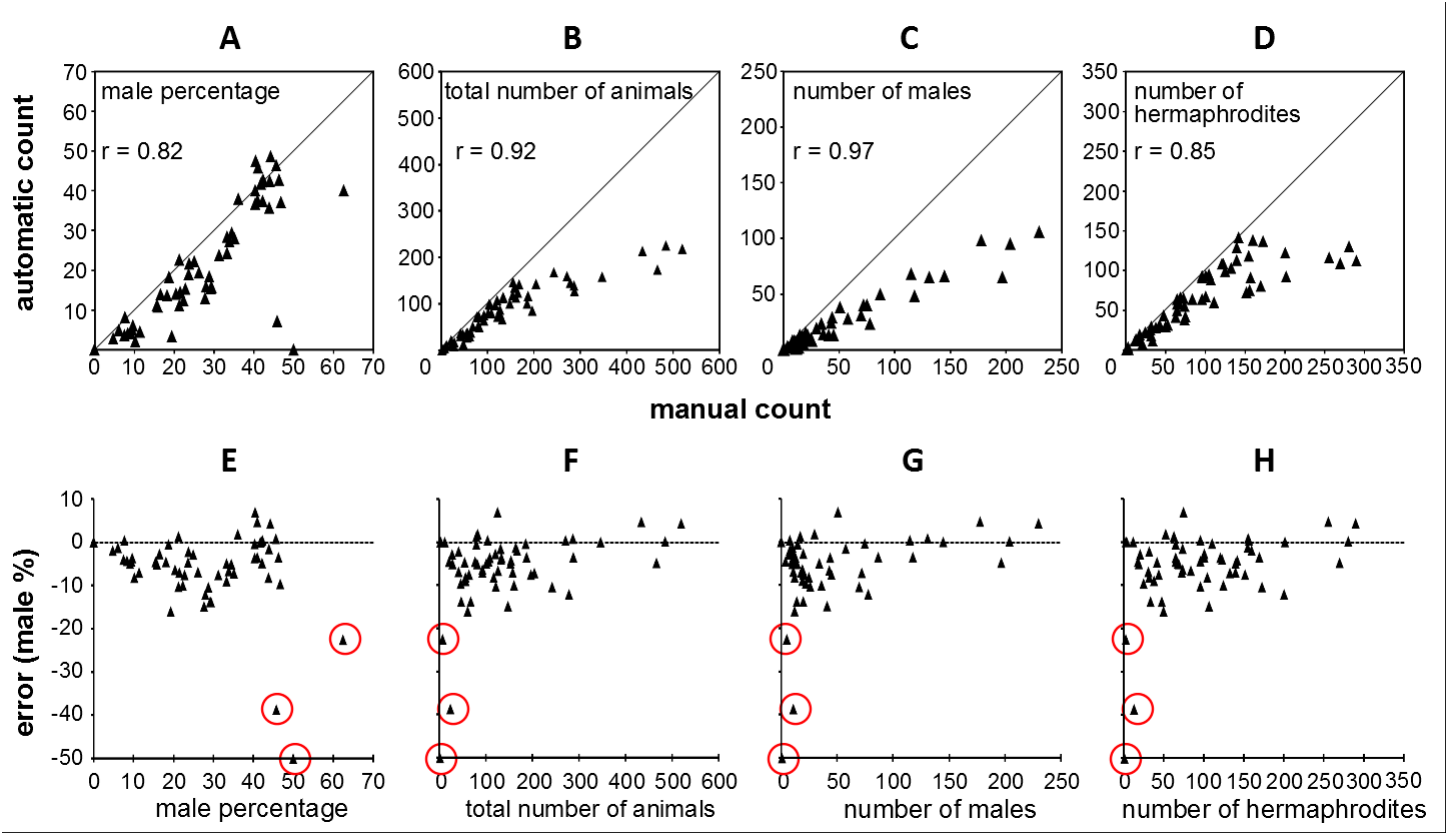
WormGender accuracy. Top, comparison of manual and automatic measurements in male percentage (A), animal count (B), male count (C) and hermaphrodite count (D). Bottom, error (difference between male % counted manually and male % counted automatically) distribution in relation to male percentage (E), animal count (F), male count (G) and hermaphrodite count (H). Raw data are listed in S2 Table.

To estimate the accuracy of WormGender in real experiments, we prepared worm plates and analyzed the same plates with both WormGender and manual count. We found that WormGender provided reliable estimate of *C*. *elegans* sex ratio. The sex ratio measured by WormGender was highly correlated to those measured by manual count (Fig 4A, r = 0.82, *p* < 0.001). The absolute error of sex ratio (difference between male percentages calculated from manual and automatic count) was reasonably small, with over 80% cases having an error lower than 10% (Fig 4E–4H).

WormGener accuracy was heavily affected by the number of animals on the plate. The absolute error of sex ratio was independent of the male percentage (Fig 4E), if we excluded wells with fewer than 10 animals (r = 0.04, *p* = 0.79). However, the absolute error was dependent on the total number of animals on the plate (r = 0.34, *p* = 0.01), with higher errors occurring at low animal counts (Fig 4F). The extreme errors occurred when total number of animals per well was very small (< 10 animals). For example, if we have only two animals in the well, a single missed animal can result in 50% error in sex ratio. Therefore, it is important to use sample sizes bigger than 10 animals per well.

The effects of too many animals per well were less critical for the accuracy of WormGender in sex ratio measurement. The number of animals counted automatically by WormGender was generally lower than the number of animals counted manually for both sexes (Fig 4B–4D). The undercounting was especially pronounced in cases where total number of animals was higher than 150 animals per well (on a 6-well plate). In those cases, due to overcrowding, some animals clustered together and became unrecognizable to the software. However, the sex ratio measurements remained accurate (Fig 4A) because the recognized worms were randomly sampled from the population and thus maintained the same sex ratio as the whole population. That is, although the software undercounted the number of animals, this impact of undercounting was similar for both male and hermaphrodite counts, therefore the sex ratio calculation remained correct. Overall, our data suggested that WormGender can accurately measure sex ratio, however it is not optimized for counting animals.

### Quantification of the *him* phenotype in mutants

It was reported that over 100 genes showed the *him* phenotype in a genome-wide RNAi screen based on manual observation [6]. About 30 of these genes have homozygous viable mutants available at the CGC. To demonstrate the utility of WormGender, we obtained 27 of these mutants and quantified the severity of their sex ratio phenotypes using WormGender. The WormGender automatic count data showed that the male percentage of these mutant strains varied widely from 0.1 to 36.46% (Table 2). Significant difference in male percentage was observed between wild-type and mutants of nine genes: *brc-1*, *C30G12*.*6*, *cep-1*, *coh-3*, *him-3*, *him-5*, *him-8*, *skr-1*, *unc-86* (Table 2).

**Table 2:** Male percentage varies in different mutant strains. Automatic counts were based on pooled data from 6 to 17 independent trials for mutants and 60 trials for wild-type; manual counts were based on pooled data from 4 to 5 independent trials. n, total number of animals.

	Automatic Count			Manual Count		
Genotype	Male %		n	Male %		n
wild-type	0.23		28185	0.35		4790
*brc-1(tm1145)*	7.86	*	4428	6.35	*	3463
*C30G12*.*6(ok2389)*	1.59	*	5297	2.30	*	4351
*cep-1(lg12501)*	3.20	*	5712	4.59	*	4050
*cki-2(ok21050)*	0.23		3532	0.44		3407
*coh-3(gk112)*	0.99	*	3130	1.58	*	1893
*F52H3*.*4(ok2692)*	0.11		1842	0.20		3422
*fbf-2(q738)*	0.47		4478	0.15		4121
*fox-1(e2643)*	0.38		6386	0.29		3424
*gpr-2(ok1179)*	0.43		5778	0.30		4047
*hcp-2(ok1757)*	0.20		2492	0.58		1560
*her-1(n695)*	0.42		5457	1.63	*	2767
*him-3(e1256)*	13.54	*	3663	16.05	*	1676
*him-5(e1490)*	36.46	*	3999	36.04	*	2486
*him-8(e1489)*	30.08	*	8113	31.09	*	1933
*klp-10(ok704)*	0.21		3286	0.11		4596
*sdc-1(n485)*	0.49		3447	0.13		2267
*sel-10(n1077)*	0.23		4686	0.05		2040
*set-2(n4589)*	0.14		4239	0.00		2014
*sgo-1(tm2443)*	0.22		5960	0.25		4463
*skr-1(ok1696)*	0.71	*	3815	3.00	*	3436
*smk-1(mn156)*	0.27		3377	2.24	*	1382
*srgp-1(ok300))*	0.24		2519	0.10		3109
*sur-7(ku119)*	0.49		2859	0.38		1572
*T08D2*.*7(ok431)*	0.42		6174	0.27		4085
*unc-86(e1416)*	2.42	*	5985	3.16	*	1394
*W02D9*.*3(ok2857)*	0.43		3038	0.26		4959
*zhp-3(ok1993)*	0.10		4154	0.11		4737

*, *p* < 0.0001 in comparison with wild-type male percentage, Chi-square test.

To confirm the performance of WormGender, we also performed manual count of male percentage in these strains, although with smaller sample sizes due to the labor-intensive nature of the assay. The manual results were highly consistent with the automatic counts from WormGender. Manual counts confirmed the nine strains that were significantly different than wild-type in WormGender measurements (Table 2). Two additional strains, *her-1* and *smk-1*, were also found to have significantly higher male percentages than wild-type in manual counts, although the difference was small in these two cases (less than 2%) (Table 2). The maximum difference of male percentages measured automatically and manually is 2.5% among all strains (Table 2). Male percentages from manual and automatic data are highly correlated with a Pearson correlation coefficient of 0.995.

Our male percentages were slightly higher than previously reported [21] for a few mutants. For example, *him-3(e1256)* had 13.54% males in our WormGender assay and 16.05% males in our manual assay (Table 2), whereas 10.90% males were reported previously [21]. This difference was likely because our assays were conducted at 25°C and previous reported experiments [21] were performed at 20°C. Male percentages based on our own manual counts from multiple independent experiments carried out at 25°C were consistent with those based on automatic counts performed at the same temperature (Table 2), confirming the utility of WormGender for sex ratio measurements.

## Conclusions

We developed the WormGender software to automatically measure sex ratio in a *C*. *elegans* population. The software measurements were similar to those from manual counts. Therefore, WormGender can serve as a useful tool for automatic measurement of *C*. *elegans* sex ratio.

## Supporting Information

S1 Table Raw data for Fig 3. (XLSX)

S2 Table Raw data for Fig 4. (XLSX)
